# Blocking the Sphingosine-1-Phosphate Receptor 2 (S1P_2_) Reduces the Severity of Collagen-Induced Arthritis in DBA-1J Mice

**DOI:** 10.3390/ijms252413393

**Published:** 2024-12-13

**Authors:** Ju-Hyun Lee, Jung-Eun Lee, Dong-Soon Im

**Affiliations:** 1 Department of Biomedical and Pharmaceutical Sciences, Graduate School, Kyung Hee University, Seoul 02446, Republic of Korea; ljh0620@khu.ac.kr (J.-H.L.); xkdnj1005@khu.ac.kr (J.-E.L.); 2 Department of Basic Pharmaceutical Sciences, Graduate School, Kyung Hee University, Seoul 02446, Republic of Korea

**Keywords:** sphingosine 1-phosphate, S1P_2_, rheumatoid, arthritis, JTE-013

## Abstract

The amount of sphingosine 1-phosphate (S1P) found in the synovial tissue of individuals with rheumatoid arthritis is five times greater than that in those with osteoarthritis. Our study aims to determine whether inhibiting S1P_2_ can mitigate collagen-induced rheumatoid arthritis (CIA) by using an S1P_2_ antagonist, JTE-013, alongside DBA-1J *S1pr2* wild-type (WT) and knock-out (KO) mice. CIA causes increases in arthritis scores, foot swelling, synovial hyperplasia, pannus formation, proteoglycan depletion, cartilage damage, and bone erosion, but these effects are markedly reduced when JTE-013 is administered to *S1pr2* WT mice. CIA also elevates mRNA expression levels of pro-inflammatory Th1/Th17 cytokines in the foot and spleen, which are significantly decreased by JTE-013 in *S1pr2* WT mice. Additionally, CIA raises Th1/Th17 and Treg cell counts, while JTE-013 reduces these elevations in the spleens of *S1pr2* WT mice. Treatment with JTE-013 or the absence of *S1pr2* curtails the differentiation of naïve T cells into Th1 and Th17 cells in a dose-dependent manner. In SW982 human synovial cells, JTE-013 lowers LPS-induced increases in pro-inflammatory cytokine levels. Overall, these findings propose that blocking S1P_2_ in immune and synovial cells may alleviate rheumatoid arthritis symptoms and offer a potential therapeutic approach.

## 1. Introduction

Autoimmune inflammatory rheumatoid arthritis (RA) is characterized by inflammation of the synovium, swelling of the joints, and damage to the cartilage [1]. RA, a chronic and serious immune disorder, affects about 0.8% of the adult population globally [2]. The pathogenesis of RA is marked by the hyperplasia of synovial lining cells (synovial fibroblasts); the formation of pannus; and the infiltration of various immune cells, including B cells, granulocytes, monocytes/macrophages, natural killer cells, and T cells [3]. Increased levels of pro-inflammatory cytokines such as interleukin (IL)-1β, IL-6, and tumor necrosis factor (TNF)-α in the synovial fluid of RA patients can lead to the production of matrix metalloproteinases, contributing to the deterioration of joint cartilage and bone [4].

Sphingosine-1-phosphate (S1P) serves as a crucial intercellular lipid mediator in autoimmune rheumatic conditions [5]. The concentration of S1P in the synovial fluid of RA patients is five times greater than that found in individuals with osteoarthritis [6]. In RA patients, peripheral blood B lymphoblastoid cell lines exhibit higher mRNA expression levels of sphingosine kinase 1, increased basal sphingosine kinase activity, and elevated S1P levels compared to matched controls [7]. Additionally, sphingosine kinase 2 is prominently expressed in the synovial fibroblasts of individuals with RA, affecting the knee joint [8]. Inhibition of sphingosine kinases, which convert sphingosine to S1P, results in decreased levels of IL-1β, IL-6, monocyte chemotactic protein-1, metalloproteinase-9, and TNF-α in peripheral blood mononuclear cells [6]. In a collagen-induced arthritis (CIA) model, silencing sphingosine kinase 1 expression via siRNA significantly lowered serum S1P levels, reduced disease severity, and curtailed both articular inflammation and joint destruction [9].

As a pro-inflammatory factor in RA, S1P produced in the synovium contributes to disease progression [10]. The immunomodulatory drug FTY720 (fingolimod), which modulates S1P_1/3/4/5_ receptors, was originally developed for treating multiple sclerosis [11]. In studies, FTY720 has been shown to inhibit arthritis in SKG mice by reducing prostaglandin E_2_ production in synovial cells and significantly suppressing arthritis both in a murine β-glucan laminarin-induced RA model and in adjuvant-induced arthritis [12,13]. Human fibroblast-like synoviocytes express S1P_1_, S1P_2_, and S1P_3_ receptors. S1P promotes synoviocyte migration via S1P_1_ and S1P_3_, and stimulates cytokine secretion through S1P_2_ and S1P_3_ [14]. Moreover, S1P_1_ enhances the expression of cyclooxygenase-2 and prostaglandin E_2_ induced by IL-1β and TNF-α in synoviocytes from RA patients and MH7A cells, a human synovial cell line [15]. S1P also causes overexpression of cyclooxygenase-2 and prostaglandin E_2_ in human articular chondrocytes [16]. The S1P_1_ modulator IMMH001 and the S1P_1_ antagonist TASP0277308 have been found to regulate CIA and adjuvant-induced arthritis [17,18]. Higher expression levels of S1P_3_ are observed in arthritic synovia compared to intact synovia and primary cultured fibroblast-like synoviocytes in arthritic joints [19]. S1P_2_ is involved in promoting osteoclastogenesis and bone resorption by influencing the PI3K, p38-MAPK, and NF-κB signaling pathways [20]. Although S1P_2_ expression in the synovium has been identified, its role in RA has not been fully explored. This study intends to examine whether blocking S1P_2_ with the antagonist JTE-013 can mitigate CIA using DBA-1J *S1pr2* wild-type (WT) and knock-out (KO) mice.

## 2. Results

### 2.1. Both JTE-013 Treatment and Deficiency of S1pr2 Inhibited the Progression of Arthritis and the Thickening of the Feet in DBA-1J Mice

**Figure 1:**
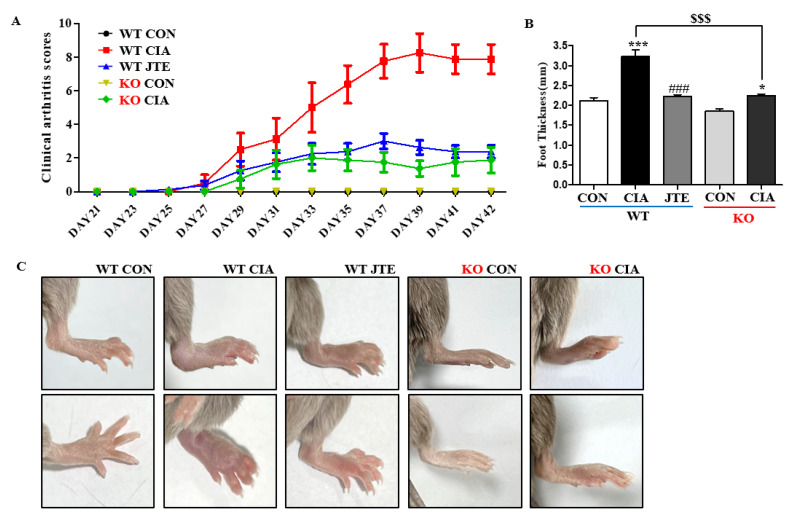
JTE-013 reduced the CIA-induced increase in arthritis score and paw thickness in *S1pr2* DBA-1J WT mice. Male *S1pr2* WT and KO DBA-1J mice, each aged 7 to 9 weeks, were injected via the tails with 100 μL of complete Freund’s adjuvant (CFA) emulsion on day 0. A booster injection of incomplete Freund’s adjuvant (IFA) was given on day 21. JTE-013, at a dose of 3 mg/kg body weight, was administered via intraperitoneal injection 30 min prior to the emulsion injection. The treatment with JTE-013 began on day 21 and continued through day 41. (**A**) Arthritis scores from day 21 to day 41 in *S1pr2* WT and KO mice. The mice were assessed for arthritis severity every other day using a specific scoring system. The criteria for scoring were: 0 for no arthritis symptoms, 1 for swelling and/or redness of one paw or digit, 2 for involvement of two joints, 3 for more than two joints being affected, and 4 for severe arthritis impacting the entire paw and digits. Each mouse’s arthritis index score was calculated by summing the scores from all individual paws. (**B**) Final paw thickness on day 42 in *S1pr2* WT and KO mice. (**C**) Representative foot images from *S1pr2* WT and KO mice. Data are presented as mean± SEM (n = 8). *** *p* < 0.001, * *p* < 0.05 compared to the control group, ^###^ *p* < 0.001 compared to the CIA group, ^$$$^ *p* < 0.001 compared to the CIA group in *S1pr2* WT mice.

To explore the therapeutic potential of S1P_2_ in CIA, macroscopic clinical features were assessed starting on the 21st day following the IFA injection in DBA-1J *S1pr2* WT mice (Figure 1A). From day 29, the arthritis score, which indicated paw swelling, showed a significant increase, peaking around day 40. JTE-013 administration resulted in a notable reduction in the arthritis score from day 33 onwards compared to the CIA group, indicating a significant decrease in foot edema (Figure 1A). On day 42, the final paw thickness was recorded before sacrifice (Figure 1B). In the CIA group, paw thickness was significantly greater than in the control group, whereas JTE-013 administration significantly reduced the CIA-induced increase in foot thickness (Figure 1A,B). Figure 1C illustrates the visibly swollen feet in the CIA groups and the reduction in swelling in JTE-013-treated mice. In DBA-1J *S1pr2* KO mice, the arthritis score increased significantly but was lower than the CIA group in *S1pr2* WT mice (Figure 1A), resembling the results observed in JTE-013-treated *S1pr2* WT mice (Figure 1A,B). Macroscopic views also confirmed that the CIA groups of *S1pr2* KO mice had significantly increased foot thickness, but significantly less swollen feet than the WT mice (Figure 1C), suggesting that JTE-013 suppresses the CIA-induced increase in arthritis score and foot thickening by inactivating S1P_2_.

### 2.2. Either JTE-013 Treatment or a Deficiency of S1pr2 Reduced the Histological Alterations Associated with RA in DBA-1J Mice

**Figure 2:**
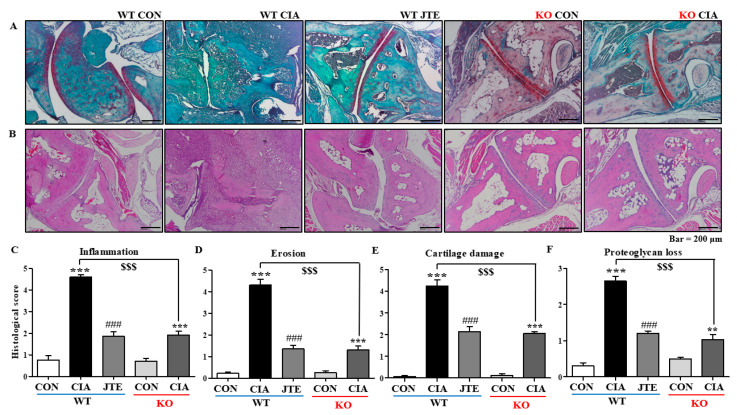
JTE-013 diminished CIA-induced arthritic changes in *S1pr2* WT mice. (**A**) H&E staining (100×) of the ankle joint in *S1pr2* WT and KO mice. (**B**) Safranin O (100×) of the ankle joint in *S1pr2* WT and KO mice. Histologic scores based on (**C**) inflammation, (**D**) bone erosion, (**E**) cartilage damage, and (**F**) proteoglycan loss in *S1pr2* WT and KO mice. Data are shown as mean ± SEM (*n* = 8). *** *p* < 0.001, ** *p* < 0.01 compared to the control group, ^###^ *p* < 0.001 compared to the CIA group, ^$$$^ *p* < 0.001 compared to the CIA group in *S1pr2* WT mice.

Histological examinations using H&E staining were conducted to evaluate inflammation and bone erosion. Synovial hyperplasia, a thickening of the synovium characteristic of RA, was evident in the CIA group of *S1pr2* WT mice (Figure 2A). As the synovial tissue expands, it forms a structure called a ‘pannus’, which damages cartilage and bones surrounding the joint. The formation of pannus tissue was more prominent in the CIA group of *S1pr2* WT mice (Figure 2A). However, treatment with JTE-013 reduced synovial hyperplasia and pannus formation in these mice (Figure 2A). Inflammation levels were quantified and depicted as bar diagrams (Figure 2C). CIA caused a significant increase in inflammation scores, while JTE-013 treatment attenuated this increase in *S1pr2* WT mice (Figure 2C). Bone erosion was similarly quantified and is shown in bar diagrams (Figure 2D), with CIA significantly raising bone erosion scores, which were mitigated by JTE-013 in *S1pr2* WT mice (Figure 2D). In *S1pr2* KO mice, the typical RA features of pannus formation, bone erosion, and synovial hyperplasia were increased in the CIA group, but to a lesser extent than in *S1pr2* WT mice (Figure 2A). This indicates that inflammation and bone erosion scores, while elevated due to CIA induction in *S1pr2* KO mice, were less severe compared to *S1pr2* WT mice (Figure 2C,D). These findings suggest that JTE-013 mitigates CIA-induced histological changes through the inactivation of S1P_2_.

**Figure 3:**
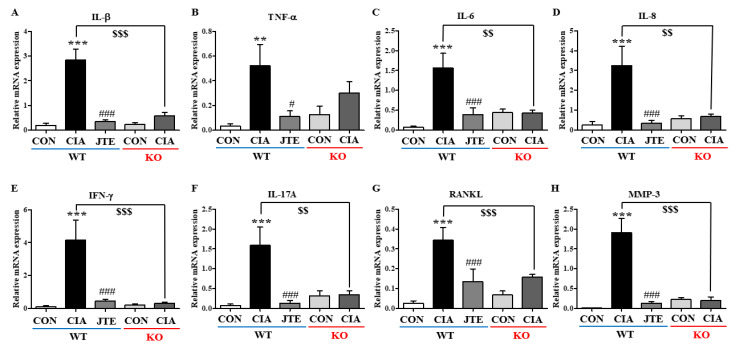
JTE-013 reduced the CIA-induced elevation of inflammatory cytokine levels in the foot of *S1pr2* WT mice. Cytokine mRNA levels were normalized to *Gapdh* mRNA level. (**A**) *Il-1b*, (**B**) *Tnf-a*, (**C**) *Il-6*, (**D**) *Il-8*, (**E**) *Ifn-g*, (**F**) *Il-17a*, (**G**) *Rankl*, and (**H**) *Mmp-3* in *S1pr2* WT and KO mice. Significance levels: *** *p* < 0.001, ** *p* < 0.01 compared to the control group; ^###^ *p* < 0.001, ^#^ *p* < 0.05 compared to the CIA group; ^$$$^ *p* < 0.001, ^$$^ *p* < 0.01 compared to the CIA group in *S1pr2* WT mice.

Additionally, Safranin O staining was used to assess cartilage damage and proteoglycan loss. Proteoglycan levels, a critical component of the articular cartilage extracellular matrix, were indicated by red staining (Figure 2B). As illustrated in Figure 2B, cartilage damage was more prevalent in the CIA group compared to controls, but JTE-013 treatment partially alleviated the damage. The extent of cartilage damage was assessed and is represented in bar diagrams in Figure 2E. While CIA significantly heightened cartilage damage, JTE-013 reduced this increase in *S1pr2* WT mice (Figure 2E). In comparison to the control group, proteoglycan loss was pronounced in the CIA group; however, JTE-013 administration offered partial protection against proteoglycan loss in *S1pr2* WT mice (Figure 2B,F). In *S1pr2* KO mice, CIA significantly induced cartilage damage and proteoglycan loss compared to control mice. However, the degree was similar to that observed in JTE-013-treated *S1pr2* WT mice (Figure 3B,E,F). These findings further imply that JTE-013 provides protection against CIA-induced cartilage damage and proteoglycan loss through S1P_2_ inactivation.

### 2.3. Either JTE-013 Treatment or Deficiency of S1pr2 Reduced mRNA Expression Levels of Pro-Inflammatory Cytokines in Foot Tissues in DBA-1J Mice

Since RA is a systemic autoimmune disease, the changes in mRNA expression levels of pro-inflammatory cytokines were assessed in foot tissues collected on the 42nd day. In the CIA group, the mRNA levels of pro-inflammatory Th1 cytokines (*Il-1β*, *Tnf-α*, *Il-6*, *Il-8*, and *Ifn-γ*) and Th17 cytokine (*Il-17a*) were elevated compared to the control group (Figure 3A–F). However, JTE-013 administration reduced these cytokine levels in *S1pr2* WT mice (Figure 3A–F). Additionally, in the CIA group, the mRNA levels of *Rankl*, which stimulates osteoclasts for bone resorption, and *Mmp-3*, a protease involved in joint destruction, were elevated (Figure 3G,H). JTE-013 significantly reduced these increases in *S1pr2* WT mice (Figure 3G,H). In *S1pr2* KO mice, CIA did not lead to an increase in the mRNA levels of Th1/Th17 cytokines, *Rankl*, or *Mmp-3* (Figure 3A–H), although the mRNA levels of *Tnf-α* or *Rankl* in KO mice exposed to CIA seemed higher than the ones not exposed.

### 2.4. Both JTE-013 Treatment and Deficiency of S1pr2 Suppressed Serum IgG Levels in DBA-1J Mice

**Figure 4:**
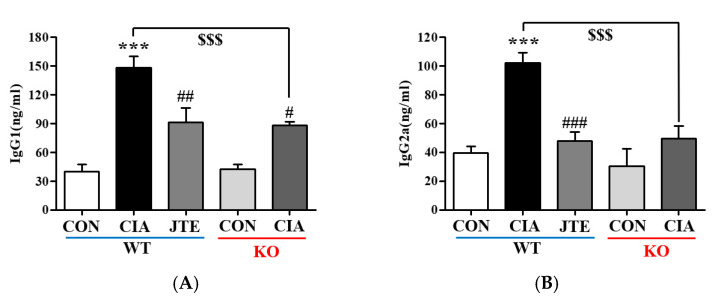
JTE-013 effectively lowered the CIA-induced elevations in serum IgG1 and IgG2a levels in *S1pr2* WT mice. Blood samples were obtained on day 42, and the serum levels of IgG1 (**A**) and IgG2a (**B**) were measured using ELISA in both S1pr2 WT and KO mice. The data are presented as mean ± SEM (n = 8). Significance levels are indicated as *** *p* < 0.001 compared to the control group; ^###^ *p* < 0.001, ^##^ *p* < 0.01, ^#^ *p* < 0.05 compared to the CIA group; ^$$$^ *p* < 0.001 compared to the CIA group in *S1pr2* WT mice.

RA involves an immune response driven by autoantibodies that mistakenly target and interact with joint antigens. IgG, the most prevalent autoantibody, is often elevated in RA. Various IgG subclasses contribute to immune and phagocytosis functions and promote inflammation. The levels of IgG1 and IgG2a subclasses, which are crucial for complement activation, were measured. In the CIA group, both IgG1 and IgG2a levels were significantly higher than in the control group; however, JTE-013 significantly lowered these levels in *S1pr2* WT mice (Figure 4A,B). In *S1pr2* KO mice, CIA induction did lead to an increase in IgG1, but not IgG2a, levels (Figure 4A,B).

### 2.5. Both JTE-013 Treatment and Deficiency of S1pr2 Reduced Spleen Enlargement in DBA-1J Mice

**Figure 5:**
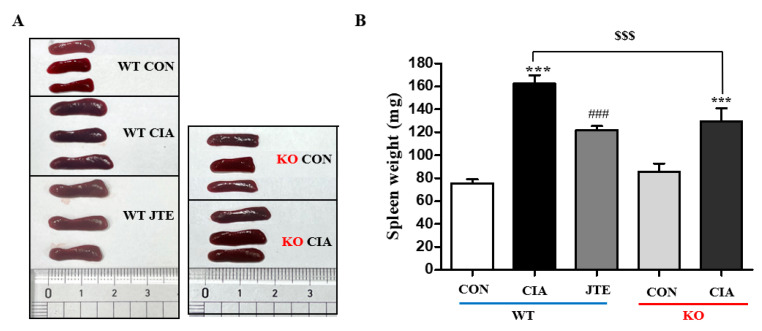
JTE-013 reduced CIA-induced spleen enlargement in *S1pr2* WT mice. (**A**) Images of spleens. (**B**) Spleen weights in *S1pr2* WT and KO mice. Results are shown as the mean ± SEM (*n* = 8). *** *p* < 0.001 compared to the control group; ^###^ *p* < 0.001 compared to the CIA group; ^$$$^ *p* < 0.001 compared to the CIA group in *S1pr2* WT mice.

The spleen, a crucial organ in the immune system, was weighed to assess changes. In the CIA group of *S1pr2* WT mice, spleen weight significantly increased compared to the control group (Figure 5A,B). JTE-013 administration markedly decreased the spleen weight gain in *S1pr2* WT mice (Figure 5A,B). In *S1pr2* KO mice, the CIA induced a significant increase in spleen weight, but the increase was significantly less than that observed in the CIA group of *S1pr2* WT mice (Figure 5A,B).

### 2.6. Both JTE-013 Treatment and Deficiency of S1pr2 Reduced CIA-Induced Elevations in Inflammatory Cytokine mRNA Expression Levels in the Spleens of DBA-1J Mice

**Figure 6:**
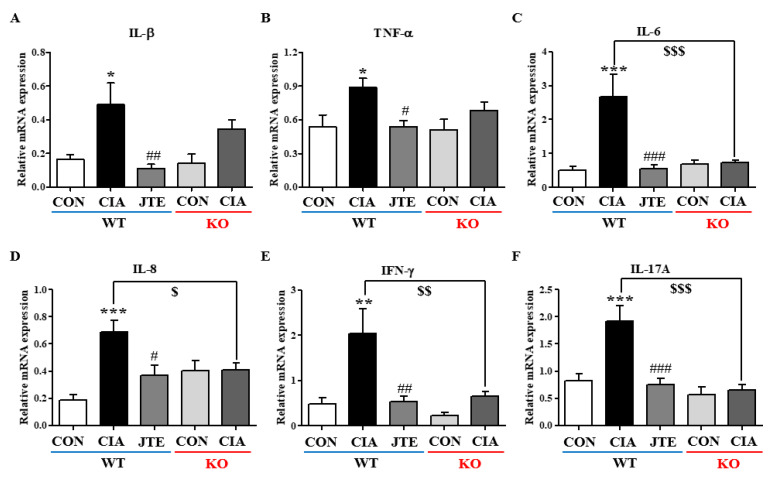
JTE-013 reduced CIA-induced increases in inflammatory cytokine levels in the spleens of *S1pr2* WT mice. Cytokine mRNA levels were quantified as ratios to *Gapdh* mRNA. (**A**) *Il-1b*, (**B**) *Tnf-a*, (**C**) *Il-6*, (**D**) *Il-8*, (**E**) *Ifn-g*, and (**F**) *Il-17a* in *S1pr2* WT and KO mice. Significance: *** *p* < 0.001, ** *p* < 0.01, * *p* < 0.05 compared to the control group; ^###^ *p* < 0.001, ^##^ *p* < 0.01, ^#^ *p* < 0.05 compared to the CIA group; ^$$$^ *p* < 0.001, ^$$^ *p* < 0.01, ^$^ *p* < 0.05 compared to the CIA group in *S1pr2* WT mice.

The analysis showed that the spleens had significantly higher mRNA expression levels of Th1 cytokines (*Il-1β*, *Tnf-α*, *Il-6*, *Il-8*, and *Ifn-γ*) and Th17 cytokine (*Il-17a*) in comparison to control mice (Figure 6A–F). These increases were significantly reduced after JTE-013 administration in *S1pr2* WT mice (Figure 6A–F). In *S1pr2* KO mice, CIA did not lead to an increase in the mRNA expression levels of pro-inflammatory cytokines in the spleen (Figure 6A–F).

### 2.7. Both JTE-013 Treatment and Deficiency of S1pr2 Diminished the CIA-Induced Rise in Populations of Th1, Th17, and Treg Cells in the Spleens of DBA-1J Mice

**Figure 7:**
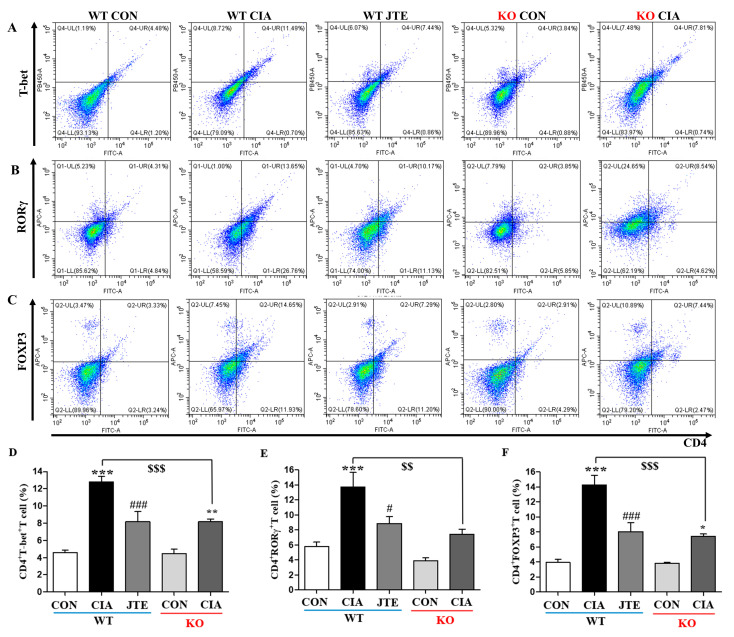
JTE-013 reduced CIA-induced increases in the populations of Th1, Th17, and Treg cells in *S1pr2* WT mice. Representative flow cytometry results of CD4^+^T-bet^+^ Th1 cells (**A**), CD4^+^T RORγt^+^ Th17 cells (**B**), and CD4^+^T FoxP3^+^ Treg cells (**C**) in *S1pr2* WT and KO mice. (**D**) Percentage of CD4^+^T-bet^+^ Th1 cells in *S1pr2* WT and KO mice. (**E**) Percentage of CD4^+^T RORγt^+^ Th17 cells in *S1pr2* WT and KO mice. (**F**) Percentage of CD4^+^T FoxP3^+^ Treg cells in *S1pr2* WT and KO mice. Significance: *** *p* < 0.001, ** *p* < 0.01, * *p* < 0.05 compared to the control group; ^###^ *p* < 0.001, ^#^ *p* < 0.05 compared to the CIA group; ^$$$^ *p* < 0.001, ^$$^ *p* < 0.01 compared to the CIA group in *S1pr2* WT mice.

The imbalance between RORγt^+^ type 17 helper T cells (Th17) and Foxp3^+^ regulatory T cells (Treg) in synovial lesions is a significant factor in the pathogenesis of rheumatoid arthritis [21,22]. Using FACS analysis, the populations of Th1, Th17, and Treg cells in the spleen were assessed, as the balance between Th1 and Th17 versus Treg cells is crucial for the progression and development of rheumatoid arthritis. CIA induction led to a significant increase in the populations of CD4^+^T-bet^+^ Th1 cells, CD4^+^RORγt^+^ Th17 cells, and CD4^+^FoxP3^+^ Treg cells, but JTE-013 mitigated this increase in *S1pr2* WT mice (Figure 7A–C). In *S1pr2* KO mice, CIA induction significantly increased the populations of Th1 and Treg cells, which were lower than those in *S1pr2* WT mice (Figure 7A,C). There was no significant increase in Th17 cells due to CIA induction in *S1pr2* KO mice (Figure 7B).

### 2.8. JTE-013 Treatment or a Lack of S1pr2 Inhibits the Differentiation of Naïve T Cells into Th1 and Th17 Cells

**Figure 8:**
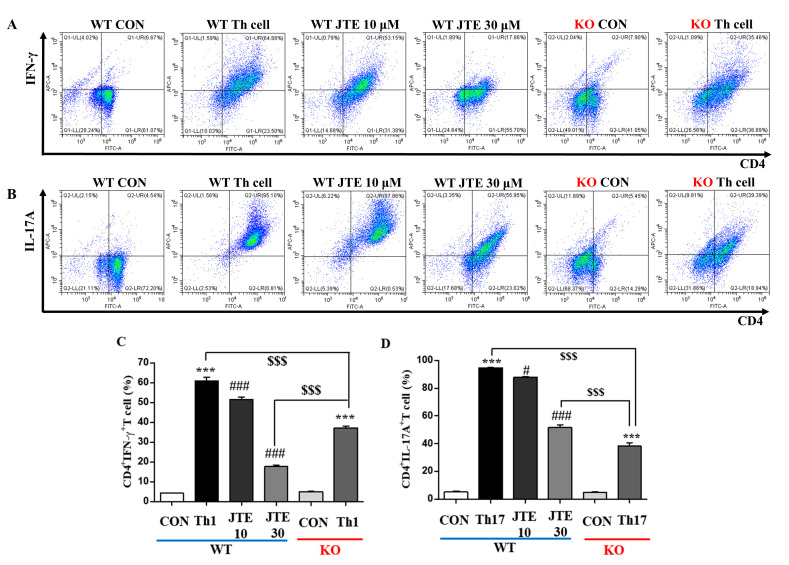
Suppressive effect of JTE-013 on T cell differentiation into Th1 and Th17 cells. CD4^+^ T cells, isolated from splenocytes, were cultured in media for Th1 or Th17 cell differentiation for 5 days in plates pre-coated with an antibody to mouse CD3. Representative flow cytometry results for CD4^+^IFN-γ^+^ Th1 cell differentiation (**A**) and CD4^+^IL-17A^+^ Th17 cell differentiation (**B**). Histograms show the percentage of CD4^+^IFN-γ^+^ cells (**C**) and CD4^+^IL-17A^+^ cells (**D**) (*n* = 5). Significance: *** *p* < 0.001 compared to the control group; ^###^ *p* < 0.001, ^#^ *p* < 0.05 compared to the CIA group; ^$$$^ *p* < 0.001 compared to the CIA group in *S1pr2* WT mice.

RA is largely driven by Th1 and Th17 cells, which play crucial roles in its pathogenesis [23,24,25]. Given our observations of significantly elevated cytokine levels in foot and spleen tissues that were suppressed by JTE-013, we investigated whether JTE-013 affects the differentiation of splenic CD4^+^ naïve T cells into Th1 and Th17 cells. After culturing naïve T cells in differentiation media specific for each type of helper T cell and conducting FACS analysis, we identified Th1 differentiation by an increase in the CD4^+^IFN-γ^+^ T cell population and Th17 differentiation by an increase in the CD4^+^IL-17A^+^ T cell population, as depicted in Figure 8A,B. JTE-013 treatment decreased differentiation into Th1 and Th17 cells in a dose-dependent manner (Figure 8A,B). From naïve T cells of *S1pr2* KO mice, differentiation media made significant populations of Th1 and Th17 cells. However, a lack of *S1pr2* also blunted the increases in the populations of Th1 and Th17 cells, more severely in Th17 differentiation than in Th1 differentiation (Figure 8A,B). These results suggest that JTE-013 administration may hinder the differentiation of naïve T cells into Th1 and Th17 inflammatory T cells, leading to reduced inflammatory cytokine release and a consequent reduction in arthritis symptoms.

### 2.9. JTE-013 Treatment Reduced the mRNA Expression Levels of Inflammatory Cytokines in SW982 Human Synovial Cells

**Figure 9:**
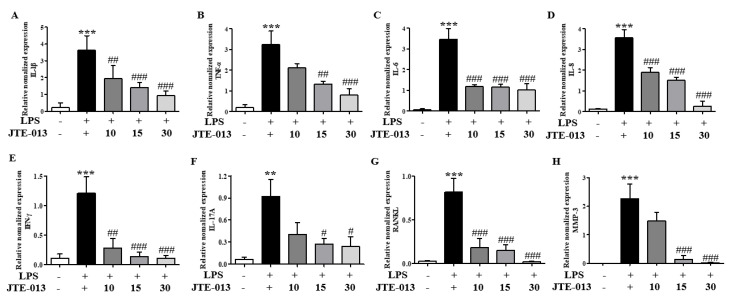
JTE-013 inhibited LPS-induced increases in pro-inflammatory cytokine mRNA expression in SW982 cells. SW982 cells were seeded at 1 × 10^5^/mL. After 24 h, JTE-013 (10, 15, 30 mM) was added, followed by LPS (100 ng/mL) incubation for 30 min. qPCR confirmed mRNA expression levels of inflammatory cytokines in SW982 cells. (**A**) IL-1β, (**B**) TNF-α, (**C**) IL-6, (**D**) IL-8, (**E**) IFN-g, (**F**) IL-17A, (**G**) RANKL, and (**H**) MMP-3. Results are shown as mean ± SEM (*n* = 5). *** *p* < 0.001, ** *p* < 0.01 vs. the control group. ^#^ *p* < 0.05, ^##^ *p* < 0.01, ^###^ *p* < 0.001 vs. the LPS-induced group.

The hyperplastic synovial membrane, a hallmark of rheumatoid arthritis, secretes cytokines that contribute to cartilage damage and likely play a role in the chronic nature of the disease. To evaluate whether JTE-013 can modulate inflammatory responses in the synovial membrane, the human synovial cell line SW982 was used. Inflammatory cytokine mRNA levels in LPS-stimulated SW982 cells were measured using qRT-PCR. LPS exposure increased the expression levels of several inflammatory cytokines (IL-1β, TNF-α, IL-6, IL-8, IFN-γ, IL-17a, RANKL, and MMP-3), but JTE-013 significantly reduced these levels in a concentration-dependent manner (Figure 9A–H).

## 3. Discussion

Numerous preclinical studies have indicated that targeting S1P production and the S1P_1_ receptor may benefit patients with RA, systemic lupus erythematosus, and systemic sclerosis by decreasing lymphocyte trafficking [5]. In 2006, the regulation of S1P and S1P_1_ signaling was suggested as a novel therapeutic target for RA based on findings of increased levels of sphingosine kinase 1, S1P, and S1P_1_ in RA synovium [15]. Initially, FTY-720 treatment was shown to suppress arthritis in both adjuvant-induced arthritis and a murine β-glucan laminarin-induced RA model [12,13]. Subsequently, the S1P_1_ modulator IMMH001 and the S1P_1_ antagonist TASP0277308 demonstrated therapeutic efficacy in CIA and adjuvant-induced arthritis models [17,18]. The potential of S1P_3_ as a therapeutic target for RA was evaluated in a CIA model using *S1pr3*-deficient mice, where these mice exhibited significantly lower clinical and histological scores, as well as reduced synovial IL-6 expression, compared to WT mice [19]. In the current study, the role of S1P_2_ in RA was explored for the first time using *S1pr2*-deficient mice and JTE-013 treatment. The study revealed several functions of S1P_2_ in the CIA model: first, S1P_2_ aggravated inflammatory RA in vivo; second, inhibiting S1P_2_ led to a reduction in pro-inflammatory cytokines in inflamed tissues and spleens; third, S1P_2_ inhibition led to decreased production of IgG1 and IgG2a in the serum; fourth, S1P_2_ inhibition resulted in decreased populations of Th1, Th17, and Treg cells in the spleens; fifth, S1P_2_ inhibition might suppress the differentiation of naïve T cells into Th1 and Th17 cells; and sixth, blocking S1P_2_ reduced LPS-induced cytokine expression in a concentration-dependent manner in human SW982 synovial cells. Therefore, activation of S1P_2_ signaling leads to an enhancement of the inflammatory immune responses during the development of CIA. Although S1P_2_ expression has been reported in the synovium, its functions have not been studied. The present study elucidates the pivotal roles of S1P_2_ in CIA and implies the therapeutic potential of S1P_2_.

Mature plasma cells are crucial in activating T cells within synovial tissue through the secretion of autoantibodies [26]. Previous research using the CIA model demonstrated that inhibiting sphingosine kinase 1 resulted in reduced production of anti-collagen IgG2a in the serum [6,9]. In the adjuvant-induced arthritis mice, treatment with S1P_1_ antagonist TASP0277308 reduced IgG levels [18]. However, no significant change in IgG levels was observed in *S1pr3*-deficient mice [19]. In the current study, we found that JTE-013 treatment or *S1pr2* deficiency led to a reduction in serum IgG1 and IgG2a levels. This suggests that S1P_1_ and S1P_2_ act as stimulatory signals for plasma cells that secrete autoantibodies, whereas S1P_3_ does not have this effect [18,19].

The erosive activity and high proliferation rate of fibroblast-like synoviocytes contribute significantly to the chronic inflammation observed in RA [27]. S1P is known to enhance the secretion of inflammatory cytokines such as IL-1β, TNF-α, and vascular endothelial growth factor in these cells [28]. Elevated S1P levels in the synovium may be responsible for attracting and retaining immune infiltrates [6]. In human RA, fibroblast-like synoviocytes express S1P_1_, S1P_2_, and S1P_3_ receptors [14]. S1P_1_/S1P_3_ signaling promotes the migration of these cells, while S1P_2_/S1P_3_ activation increases IL-6 and IL-8 secretion [14]. Inhibition of S1P_1/3_ has been shown to reduce the proliferation, migration, invasion, and pro-inflammatory cytokine release in MH7A cells, a human RA synovial cell line, whereas S1P_2_ suppression primarily affects cell invasion and the release of pro-inflammatory IL-1β and prostaglandin E_2_ [28]. S1P_1_ signaling in synoviocytes is closely linked to synovial hyperplasia, inflammation, and RANKL-induced osteoclastogenesis in RA [29]. The findings in human SW982 synovial cells corroborate previous studies in fibroblast-like synoviocytes and provide a strong mechanistic foundation for the therapeutic efficacy of S1P_2_ blockade, as demonstrated in the current CIA model.

The IL-23/IL-17 axis plays a crucial role in the development of RA [30]. In vitro, S1P has demonstrated a similar ability to IL-23 in terms of promoting the proliferation and IL-17-secreting activity of CD4^+^ T cells in the presence of IL-1β, TGF-β1, and IL-6 [31]. Treatment with FTY720 significantly reduced IL-17 production in Th17 cells [32]. Our findings showed that JTE-013 decreased the populations of Th17, Th1, and Treg cells in the spleens. This reduction in T cell populations might explain the suppressive effects of JTE-013 on the expression of pro-inflammatory Th1 and Th17 cytokines in the spleens and feet. Furthermore, JTE-013 treatment or *S1pr2* deficiency inhibited the differentiation of naïve T cells into Th17 and Th1 cells. The immune-enhancing role of S1P_2_ has also been observed in other disease models, such as allergic asthma, pulmonary fibrosis, and atopic dermatitis [33,34,35,36]. Thus, the present study not only discovers the functional roles of S1P_2_ in the CIA development, but also expands the immune-enhancing role of S1P_2_ to an autoimmune RA disease model. While additional research is needed, blocking S1P_2_ may lead to the inhibition of Th1 and Th17 cell differentiation; a decrease in Th1, Th17, and Treg cell populations; reduced expression of pro-inflammatory cytokines; and alleviation of RA symptoms.

## 4. Materials and Methods

### 4.1. Materials

JTE-013 was sourced from Cayman Chemicals, located in Ann Arbor, MI, USA. Bovine type II collagen, complete Freund’s adjuvant (CFA), and incomplete Freund’s adjuvant (IFA) were acquired from Chondrex Inc. in Woodinville, WA, USA. Additional chemicals were obtained from Sigma-Aldrich, based in St. Louis, MO, USA.

### 4.2. Cell Culture

Human SW982 synovial cells were procured from the American Type Culture Collection (ATCC) in Manassas, VA, USA. These cells were cultured in Dulbecco’s Modified Eagle Medium (DMEM) supplemented with 10% (*v*/*v*) heat-inactivated fetal bovine serum, 100 U/mL penicillin, and 50 μg/mL streptomycin and maintained at 37 °C within a humidified incubator containing 5% CO_2_.

### 4.3. Animals

Richard Proia at the NIH generously provided three heterozygous mice with the *S1pr2* gene [37]. These mice had been backcrossed for eight generations with DBA-1J mice from Orient, located in Seoul, Republic of Korea. The *S1pr2* wild-type (WT) and knockout (KO) DBA-1J mice were kept in the Laboratory Animal Facility at Kyung Hee University, where they had unrestricted access to food and water. Male *S1pr2* WT and KO DBA/1J mice, aged eight weeks, were utilized in this study. The animal protocol for this research was reviewed and approved by the Institutional Animal Care Committee of Kyung Hee University, ensuring ethical procedures and animal care under Approval Number KH-SASP-23-196.

### 4.4. Human SW982 Synovial Cell Treatment

In preparation for the experiments, SW982 cells were plated at a density of 1 × 10^5^ cells per well in six-well plates and allowed to culture overnight. The following day, the medium was removed, and the wells were rinsed with PBS. The cells were then treated with JTE-013 and incubated for 30 min. Lipopolysaccharide (LPS; 100 ng/mL) was subsequently added, and the cells were cultured overnight before being harvested for qRT-PCR analysis.

### 4.5. Induction of Rheumatoid Arthritis in DBA-1J Mice and JTE-013 Administration

CIA is a widely used autoimmune animal model for researching RA. Male *S1pr2* WT and KO DBA-1J mice weighing 23 to 25 g and aged from 7 to 9 weeks were randomly divided into five experimental groups (*n* = 8 per group), namely, the control *S1pr2* WT group, CIA *S1pr2* WT group, CIA+JTE-013-treated *S1pr2* WT group, control *S1pr2* KO group, and CIA *S1pr2* KO group. A CFA emulsion was prepared as previously described [38,39,40], 100 μL of which was injected into the tails of mice that had been anesthetized with Avertin on day 0 (D0). Thereafter, a booster injection of IFA was administrated on day 21. JTE-013 (3 mg/kg body weight) was administrated by i.p. injection 30 min before the emulsion injection. The JTE-013 treatment began on day 21 and continued until day 41. During this period, we evaluated the clinical scores and body weights of the mice [38,39,40].

### 4.6. Measurement of the Severity of Arthritis

Beginning on day 21, the severity of arthritis in the mice was evaluated every other day using a scoring system described in previous studies. The scoring criteria were as follows: 0 indicated no signs of arthritis, 1 represented swelling and/or redness of the paw or a single digit, 2 indicated involvement of two joints, 3 signified involvement of more than two joints, and 4 denoted severe arthritis affecting the entire paw and digits. An arthritis index score for each mouse was determined by adding together the scores from each individual paw [38,41,42].

### 4.7. Histological Assessment of Arthritis

The Korea Non-clinical Technology Solution Center (Seoul, Korea) prepared paraffin sections from the tissues of DBA-1J mouse feet. These sections were utilized to identify histological changes associated with rheumatoid arthritis through Safranin O staining using the NovaUltra Safranin O Stain Kit (cat. IW-3011, IHC WORLD, Ellicott City, MD, USA) according to the manufacturer’s guidelines. For hematoxylin and eosin (H&E) staining, the paraffin-embedded sections were first deparaffinized by immersing them in xylene for 5 min, followed by dehydration with ethanol. After hematoxylin treatment, the sections were rinsed with tap water, re-dehydrated with ethanol, stained with Eosin Y, fixed with ethanol, and finally mounted for observation using Permount (Thermo Fischer Scientific Inc, Waltham, MA, USA). Inflammation, pannus formation, and erosion of cartilage and bone were assessed using previously established indices [43]. Inflammation and bone erosion were rated on a scale from 0 to 5: 0 indicated normal; 1 represented minimal changes (local inflammatory cell infiltration or mild swelling); 2 mild changes (local inflammatory cell infiltration and mild swelling); 3 moderate resorption of trabecular and cortical bone without defects or loss; 4 marked changes (significant inflammatory cell infiltration and damage to cortical and trabecular bones); and 5 severe infiltration of inflammatory cells and complete skeletal destruction [44,45,46]. Cartilage damage was evaluated by the loss of Safranin-O staining and scored on a semi-quantitative scale from 0 to 4: 0 for intact cartilage; 1 for minor damage; 2 for moderate damage; 3 for high damage; and 4 for severe damage [47,48].

### 4.8. Flowcytometric Analysis

To assess the T cell population, single cells extracted from spleens were stained with an FITC-conjugated rat antibody targeting CD4 (catalog no. 11-0041-82, eBioscience, San Diego, CA, USA) at 4 °C for 20 min. The cells were then fixed at room temperature for one hour using an intracellular fixation buffer (catalog no. 00-8222-49, eBioscience). Following fixation, the cells were permeabilized with a permeabilization buffer (catalog no. 88-8824-00, eBioscience) and stained for one hour at room temperature with either APC-conjugated rat anti-Foxp3 (catalog no. 17-5773-82, eBioscience), eFluor 450-conjugated rat anti-T-bet (catalog no. 48-5825-82, eBioscience), or APC-conjugated rat anti-RORγt (catalog no. 17-6988-82). The analysis was conducted using a CytoFLEX Flow cytometer (Beckman Coulter, Brea, CA, USA).

### 4.9. Quantitative Real-Time PCR

To evaluate the expression of inflammatory markers in mouse feet using qRT-PCR, first-strand cDNA was synthesized from total RNA extracted with TRIzol reagent (Invitrogen, Waltham, MA, USA). Total RNA was obtained from the feet, spleens, or SW982 human synovial cells. The RNA was reverse-transcribed into cDNA using MMLV reverse transcriptase (Promega, Madison, WI, USA). qRT-PCR was performed with Thunderbird Next SYBR qPCR Mix (Toyobo, Osaka, Japan) on a CFX Connect Real-Time System (Bio-Rad, Hercules, CA, USA). Specific primers were utilized for *Mus musculus Il-1β*, *Tnf-α*, *Il-6*, *Il-8*, *Ifn-γ*, *Il-17a*, *Rankl*, *Mmp-3*, and *Gapdh*, as well as *Homo sapiens GAPDH*, *IL-1β*, *TNF-α, IL-6*, *IL-8*, *IFN-γ*, *IL-17A*, *RANKL*, and *MMP-3*. The thermal cycling conditions included an initial cycle at 95 °C for 4 min, followed by 40 cycles at 95 °C for 30 s and 57 °C for 30 s, with a final cycle at 95 °C for 30 s. Gene expression was quantified using the 2^−ΔΔCt^ method.

### 4.10. Enzyme-Linked Immunosorbent Assay (ELISA)

Mouse sera were kept at −80 °C until they were needed for analysis. The serum concentrations of IgG1 and IgG2a were measured using specific ELISA kits (eBioscience, San Diego, CA, USA; IgG1: catalog no. 88-50410-88, IgG2a: catalog no. 88-50420-88). Avidin–horseradish peroxidase was utilized for detection, and absorbance was recorded at 450 nm.

### 4.11. T Cell Differentiation

Naïve CD4^+^ T cells were isolated from mouse splenocytes using magnetic beads (Naïve CD4^+^ T Cell Isolation Kit, Miltenyi Biotec, Bergisch Gladbach, Germany). These cells were then placed in Th cell differentiation media in 24-well plates that had been coated with anti-mouse CD3 and CD28 antibodies and cultured for three days. The Th1 differentiation medium included rmIL-12, hIL-2, and anti-IL-4, while the Th17 differentiation medium contained rmIL-6, hTGF-β, anti-IFN-γ, and anti-IL-4. On the third day, fresh Th cell differentiation media was added, and the cells were cultured for an additional three days. On day six, the cells were collected 4–6 h after treatment with Golgi inhibitors and restimulation with anti-CD3, and Th cell differentiation was assessed using flow cytometry. JTE-013 at concentrations of 10 and 30 µM was added to the Th differentiation media to determine its effect.

### 4.12. Verification of Normality and Statistical Analysis

The results from the animal experiments are presented as means ± standard error of the mean (SEM) based on eight measurements. To evaluate whether the data followed a normal distribution, the Kolmogorov–Smirnov (KS) test was applied. Differences were deemed statistically significant using analysis of variance (ANOVA) followed by Tukey’s multiple comparison test, with significance set at a *p*-value < 0.05. Both the normality check and statistical analyses were conducted using GraphPad Prism software (version 10.3.1, GraphPad Software, Inc., La Jolla, CA, USA).

## 5. Conclusions

In summary, these findings propose that blocking S1P_2_ in immune and synovial cells may alleviate rheumatoid arthritis symptoms and could serve as a potential therapeutic strategy for rheumatoid arthritis.

## Data Availability

Data are available from the authors upon reasonable request.

## References

[1] Smolen J.S., Aletaha D., Barton A., Burmester G.R., Emery P., Firestein G.S., Kavanaugh A., McInnes I.B., Solomon D.H., Strand V. (2018). Rheumatoid arthritis. Nat. Rev. Dis. Primers.

[2] Finckh A., Gilbert B., Hodkinson B., Bae S.C., Thomas R., Deane K.D., Alpizar-Rodriguez D., Lauper K. (2022). Global epidemiology of rheumatoid arthritis. Nat. Rev. Rheumatol..

[3] Jang S., Kwon E.J., Lee J.J. (2022). Rheumatoid Arthritis: Pathogenic Roles of Diverse Immune Cells. Int. J. Mol. Sci..

[4] Adams S.B., Setton L.A., Bell R.D., Easley M.E., Huebner J.L., Stabler T., Kraus V.B., Leimer E.M., Olson S.A., Nettles D.L. (2015). Inflammatory Cytokines and Matrix Metalloproteinases in the Synovial Fluid After Intra-articular Ankle Fracture. Foot Ankle Int..

[5] Burg N., Salmon J.E., Hla T. (2022). Sphingosine 1-phosphate receptor-targeted therapeutics in rheumatic diseases. Nat. Rev. Rheumatol..

[6] Lai W.Q., Irwan A.W., Goh H.H., Howe H.S., Yu D.T., Valle-Oñate R., McInnes I.B., Melendez A.J., Leung B.P. (2008). Anti-inflammatory effects of sphingosine kinase modulation in inflammatory arthritis. J. Immunol..

[7] Pi X., Tan S.Y., Hayes M., Xiao L., Shayman J.A., Ling S., Holoshitz J. (2006). Sphingosine kinase 1-mediated inhibition of Fas death signaling in rheumatoid arthritis B lymphoblastoid cells. Arthritis Rheum..

[8] Kamada K., Arita N., Tsubaki T., Takubo N., Fujino T., Soga Y., Miyazaki T., Yamamoto H., Nose M. (2009). Expression of sphingosine kinase 2 in synovial fibroblasts of rheumatoid arthritis contributing to apoptosis by a sphingosine analogue, FTY720. Pathol. Int..

[9] Lai W.Q., Irwan A.W., Goh H.H., Melendez A.J., McInnes I.B., Leung B.P. (2009). Distinct roles of sphingosine kinase 1 and 2 in murine collagen-induced arthritis. J. Immunol..

[10] Lai W.Q., Melendez A.J., Leung B.P. (2010). Role of sphingosine kinase and sphingosine-1-phosphate in inflammatory arthritis. World J. Biol. Chem..

[11] Park S.J., Im D.S. (2017). Sphingosine 1-Phosphate Receptor Modulators and Drug Discovery. Biomol. Ther..

[12] Tsunemi S., Iwasaki T., Kitano S., Imado T., Miyazawa K., Sano H. (2010). Effects of the novel immunosuppressant FTY720 in a murine rheumatoid arthritis model. Clin. Immunol..

[13] Matsuura M., Imayoshi T., Chiba K., Okumoto T. (2000). Effect of FTY720, a novel immunosuppressant, on adjuvant-induced arthritis in rats. Inflamm. Res..

[14] Zhao C., Fernandes M.J., Turgeon M., Tancrède S., Di Battista J., Poubelle P.E., Bourgoin S.G. (2008). Specific and overlapping sphingosine-1-phosphate receptor functions in human synoviocytes: Impact of TNF-alpha. J. Lipid Res..

[15] Kitano M., Hla T., Sekiguchi M., Kawahito Y., Yoshimura R., Miyazawa K., Iwasaki T., Sano H., Saba J.D., Tam Y.Y. (2006). Sphingosine 1-phosphate/sphingosine 1-phosphate receptor 1 signaling in rheumatoid synovium: Regulation of synovial proliferation and inflammatory gene expression. Arthritis Rheum..

[16] Masuko K., Murata M., Nakamura H., Yudoh K., Nishioka K., Kato T. (2007). Sphingosine-1-phosphate attenuates proteoglycan aggrecan expression via production of prostaglandin E2 from human articular chondrocytes. BMC Musculoskelet. Disord..

[17] Jin J., Ji M., Fu R., Wang M., Xue N., Xiao Q., Hu J., Wang X., Lai F., Yin D. (2019). Sphingosine-1-Phosphate Receptor Subtype 1 (S1P1) Modulator IMMH001 Regulates Adjuvant- and Collagen-Induced Arthritis. Front. Pharmacol..

[18] Fujii Y., Hirayama T., Ohtake H., Ono N., Inoue T., Sakurai T., Takayama T., Matsumoto K., Tsukahara N., Hidano S. (2012). Amelioration of collagen-induced arthritis by a novel S1P1 antagonist with immunomodulatory activities. J. Immunol..

[19] Inoue T., Kohno M., Nagahara H., Murakami K., Sagawa T., Kasahara A., Kaneshita S., Kida T., Fujioka K., Wada M. (2019). Upregulation of sphingosine-1-phosphate receptor 3 on fibroblast-like synoviocytes is associated with the development of collagen-induced arthritis via increased interleukin-6 production. PLoS ONE.

[20] Hsu L.C., Reddy S.V., Yilmaz Ö., Yu H. (2019). Sphingosine-1-Phosphate Receptor 2 Controls Podosome Components Induced by RANKL Affecting Osteoclastogenesis and Bone Resorption. Cells.

[21] Nadkarni S., Mauri C., Ehrenstein M.R. (2007). Anti-TNF-alpha therapy induces a distinct regulatory T cell population in patients with rheumatoid arthritis via TGF-beta. J. Exp. Med..

[22] Behrens F., Himsel A., Rehart S., Stanczyk J., Beutel B., Zimmermann S.Y., Koehl U., Möller B., Gay S., Kaltwasser J.P. (2007). Imbalance in distribution of functional autologous regulatory T cells in rheumatoid arthritis. Ann. Rheum. Dis..

[23] Chabaud M., Fossiez F., Taupin J.L., Miossec P. (1998). Enhancing effect of IL-17 on IL-1-induced IL-6 and leukemia inhibitory factor production by rheumatoid arthritis synoviocytes and its regulation by Th2 cytokines. J. Immunol..

[24] Miossec P., Korn T., Kuchroo V.K. (2009). Interleukin-17 and type 17 helper T cells. N. Engl. J. Med..

[25] Yang P., Qian F.Y., Zhang M.F., Xu A.L., Wang X., Jiang B.P., Zhou L.L. (2019). Th17 cell pathogenicity and plasticity in rheumatoid arthritis. J. Leukoc. Biol..

[26] Takemura S., Klimiuk P.A., Braun A., Goronzy J.J., Weyand C.M. (2001). T cell activation in rheumatoid synovium is B cell dependent. J. Immunol..

[27] Masoumi M., Bashiri H., Khorramdelazad H., Barzaman K., Hashemi N., Sereshki H.A., Sahebkar A., Karami J. (2021). Destructive Roles of Fibroblast-like Synoviocytes in Chronic Inflammation and Joint Damage in Rheumatoid Arthritis. Inflammation.

[28] Wang M., Wu H., Wang R., Dai X., Deng R., Wang Y., Bu Y., Sun M., Zhang H. (2021). Inhibition of sphingosine 1-phosphate (S1P) receptor 1/2/3 ameliorates biological dysfunction in rheumatoid arthritis fibroblast-like synoviocyte MH7A cells through Gαi/Gαs rebalancing. Clin. Exp. Pharmacol. Physiol..

[29] Takeshita H., Kitano M., Iwasaki T., Kitano S., Tsunemi S., Sato C., Sekiguchi M., Azuma N., Miyazawa K., Hla T. (2012). Sphingosine 1-phosphate (S1P)/S1P receptor 1 signaling regulates receptor activator of NF-κB ligand (RANKL) expression in rheumatoid arthritis. Biochem. Biophys. Res. Commun..

[30] Li H., Tsokos G.C. (2021). IL-23/IL-17 Axis in Inflammatory Rheumatic Diseases. Clin. Rev. Allergy Immunol..

[31] Huang M.C., Watson S.R., Liao J.J., Goetzl E.J. (2007). Th17 augmentation in OTII TCR plus T cell-selective type 1 sphingosine 1-phosphate receptor double transgenic mice. J. Immunol..

[32] Liao J.J., Huang M.C., Goetzl E.J. (2007). Cutting edge: Alternative signaling of Th17 cell development by sphingosine 1-phosphate. J. Immunol..

[33] Park S.J., Im D.S. (2020). Blockage of sphingosine-1-phosphate receptor,4-dinitrochlorobenzene-induced atopic dermatitis in mice. Acta Pharmacol. Sin..

[34] Park S.J., Im D.S. (2019). Blockage of sphingosine-1-phosphate receptor 2 attenuates allergic asthma in mice. Br. J. Pharmacol..

[35] Park S.J., Im D.S. (2019). Deficiency of Sphingosine-1-Phosphate Receptor 2 (S1P_2_) Attenuates Bleomycin-Induced Pulmonary Fibrosis. Biomol. Ther..

[36] Kang J., Lee J.H., Im D.S. (2020). Topical Application of S1P_2_ Antagonist JTE-013 Attenuates 2,4-Dinitrochlorobenzene-Induced Atopic Dermatitis in Mice. Biomol. Ther..

[37] Kono M., Belyantseva I.A., Skoura A., Frolenkov G.I., Starost M.F., Dreier J.L., Lidington D., Bolz S.S., Friedman T.B., Hla T. (2007). Deafness and stria vascularis defects in S1P2 receptor-null mice. J. Biol. Chem..

[38] Park B., Lee M., Kim S.D., Jeong Y.S., Kim J.C., Yang S., Kim H.Y., Bae Y.S. (2021). Activation of formyl peptide receptor 1 elicits therapeutic effects against collagen-induced arthritis. J. Cell. Mol. Med..

[39] Courtenay J.S., Dallman M.J., Dayan A.D., Martin A., Mosedale B. (1980). Immunisation against heterologous type II collagen induces arthritis in mice. Nature.

[40] Myers L.K., Rosloniec E.F., Cremer M.A., Kang A.H. (1997). Collagen-induced arthritis, an animal model of autoimmunity. Life Sci..

[41] Liu L., Yang J., Zu B., Wang J., Sheng K., Zhao L., Xu W. (2018). Acacetin regulated the reciprocal differentiation of Th17 cells and Treg cells and mitigated the symptoms of collagen-induced arthritis in mice. Scand. J. Immunol..

[42] Deng Y., Luo H., Shu J., Shu H., Lu C., Zhao N., Geng Y., He X., Lu A. (2020). Pien Tze Huang alleviate the joint inflammation in collagen-induced arthritis mice. Chin. Med..

[43] Hayer S., Vervoordeldonk M.J., Denis M.C., Armaka M., Hoffmann M., Bäcklund J., Nandakumar K.S., Niederreiter B., Geka C., Fischer A. (2021). ‘SMASH’ recommendations for standardised microscopic arthritis scoring of histological sections from inflammatory arthritis animal models. Ann. Rheum. Dis..

[44] Corr M., Boyle D.L., Ronacher L.M., Lew B.R., van Baarsen L.G., Tak P.P., Firestein G.S. (2011). Interleukin 1 receptor antagonist mediates the beneficial effects of systemic interferon beta in mice: Implications for rheumatoid arthritis. Ann. Rheum. Dis..

[45] Kyburz D., Corr M. (2003). The KRN mouse model of inflammatory arthritis. Springer Seminars in Immunopathology.

[46] Choe J.Y., Crain B., Wu S.R., Corr M. (2003). Interleukin 1 receptor dependence of serum transferred arthritis can be circumvented by toll-like receptor 4 signaling. J. Exp. Med..

[47] Douni E., Sfikakis P.P., Haralambous S., Fernandes P., Kollias G. (2004). Attenuation of inflammatory polyarthritis in TNF transgenic mice by diacerein: Comparative analysis with dexamethasone, methotrexate and anti-TNF protocols. Arthritis Res. Ther..

[48] Pettit A.R., Ji H., von Stechow D., Müller R., Goldring S.R., Choi Y., Benoist C., Gravallese E.M. (2001). TRANCE/RANKL knockout mice are protected from bone erosion in a serum transfer model of arthritis. Am. J. Pathol..

